# The Effect of Isomaltulose Together with Green Tea on Glycemic Response and Antioxidant Capacity: A Single-Blind, Crossover Study in Healthy Subjects

**DOI:** 10.3390/nu9050464

**Published:** 2017-05-06

**Authors:** Passakorn Suraphad, Phim On Suklaew, Sathaporn Ngamukote, Sirichai Adisakwattana, Kittana Mäkynen

**Affiliations:** Department of Nutrition and Dietetics, Faculty of Allied Health Sciences, Chulalongkorn University, Bangkok 10330, Thailand; tostimulus@hotmail.com (P.S.); red_flower_bow@hotmail.com (P.O.S.); amppam10@gmail.com (S.N.); Sirichai.a@chula.ac.th (S.A.)

**Keywords:** isomaltulose, green tea, sucrose, glycemic response, antioxidant capacity

## Abstract

Isomaltulose, a naturally-occurring isomer of sucrose, is commonly used as an alternative sweetener in foods and beverages. The goal of this study was to determine the effect of isomaltulose together with green tea on postprandial plasma glucose and insulin concentration, as well as antioxidant capacity in healthy subjects. In a randomized, single-blind, crossover study, 15 healthy subjects (eight women and seven men; ages 23.5 ± 0.7 years; with body mass index of 22.6 ± 0.4 kg/m^2^) consumed five beverages: (1) 50 g sucrose in 400 mL water; (2) 50 g isomaltulose in 400 mL of water; (3) 400 mL of green tea; (4) 50 g sucrose in 400 mL of green tea; and (5) 50 g isomaltulose in 400 mL of green tea. Incremental area under postprandial plasma glucose, insulin, ferric reducing ability of plasma (FRAP) and malondialdehyde (MDA) concentration were determined during 120 min of administration. Following the consumption of isomaltulose, the incremental 2-h area under the curve (AUC_0–2 h_) indicated a higher reduction of postprandial glucose (43.4%) and insulin concentration (42.0%) than the consumption of sucrose. The addition of green tea to isomaltulose produced a greater suppression of postprandial plasma glucose (20.9%) and insulin concentration (37.7%). In accordance with antioxidant capacity, consumption of sucrose (40.0%) and isomaltulose (28.7%) caused the reduction of green tea-induced postprandial increases in FRAP. A reduction in postprandial MDA after drinking green tea was attenuated when consumed with sucrose (34.7%) and isomaltulose (17.2%). In conclusion, green tea could enhance the reduction of postprandial glucose and insulin concentration when consumed with isomaltulose. In comparison with sucrose, isomaltulose demonstrated less alteration of plasma antioxidant capacity after being consumed with green tea.

## 1. Introduction

There has been a marked increase in the consumption of sugar-sweetened beverages across the globe [1,2]. Recent evidence has been able to substantiate the relationship between the consumption of sugar-sweetened beverages and the risks of type 2 diabetes, obesity and cardiovascular diseases [3]. A number of randomized clinical trials have reported that the consumption of sweetened beverages increased body weight and fat mass after 10 weeks [4]. There has been increasing concern regarding the health effects of being overweight and obesity in adults, and this has, therefore, led to a rising demand for low-energy food products. Low-glycemic sweeteners commonly offer an alternative approach to using caloric sugars as substitutes for sucrose and high fructose corn syrup in foods and beverages [5,6,7].

Isomaltulose (6-*O*-d-glucopyranosyl-d-fructose), one type of low-glycemic sweetener, is a naturally-occurring disaccharide found in honey, sugarcane and molasses [8]. Like sucrose, it is digested by α-glucosidase in the small intestine and contributes the same caloric value of approximately 4 kcal/g [9,10]. However, the rates of isomaltulose digestion and absorption are much slower than sucrose [11,12]. Furthermore, the consumption of isomaltulose was found to be safe without gastrointestinal side effects [8]. Clinical studies provided more convincing evidence in support of isomaltulose for controlling postprandial glucose profile in humans [12,13,14]. Therefore, isomaltulose has been applied most recently in ready-to-drink products, such as sports drinks, instant drinks and milk-based drinks [10,15].

Green tea (*Camellia sinensis*), one of the most popular beverages in the world, is a rich source of polyphenols, specifically, epicatechin (EC), epicatechin gallate (ECG) and epigallocatechin gallate (EGCG) [16]. Evidence from cohort studies showed an association between higher intake of tea-derived polyphenols, such as flavan-3-ols [17,18] and, more specifically, catechins and theaflavins [19], and lower risk of impaired glucose metabolism. However, no correlation was found for other polyphenol groups [20]. Several studies have shown the beneficial effects of green tea, including anti-hyperglycemic, anti-oxidative, anti-carcinogenic, anti-inflammatory and hypocholesterolemic activity [21,22,23]. For example, catechin-rich green tea improves postprandial glucose concentration and plasma antioxidant capacity in human subjects [24,25]. In a clinical trial, consumption of 1.5 g green tea extracts in 500 mL water (total polyphenols with approximately 500 mg gallic acid equivalent) with 75 g glucose decreased postprandial plasma glucose concentration in healthy subjects [26]. In a randomized crossover design, consumption of 50 g of white bread with a green tea beverage (5 g green tea in 200 mL) significantly decreased the postprandial plasma glucose level in healthy subjects [27]. Furthermore, sucrose-loading (2.0 g/kg body weight) with green tea extract (0.5 g/kg body weight) significantly decreased plasma glucose level in rats [28]. According to the literature, the plasma lowering effect of green tea was evidently supported by clinical studies in human subjects who consumed green tea with calorie sugars. However, there have not been any reports examining whether consumption of isomaltulose and green tea alters plasma glucose concentration and antioxidant status. Therefore, the aim of the current study was to investigate the effect of isomaltulose together with green tea on postprandial plasma glucose and insulin concentration and antioxidant capacity in healthy subjects.

## 2. Materials and Methods 

### 2.1. Preparation of Green Tea Beverages 

Instant green tea leaf products were purchased from the local market. The content of the total amount of polyphenolic compounds was determined using the Folin–Ciocalteu method, according to the ISO 14502-1 method [29]. The phytochemical analysis of catechins was determined by using high-performance liquid chromatograph (HPLC), according to the ISO 14502-2 method [30]. The polyphenol content of the instant green tea was 12.30 g/100 g (dry weight basis). The phytochemical components were 2.16 g (−)-epigallocatechin, 1.85 g (−)-epigallocatechin gallate, 0.7 g (−)-epicatechin, 0.57 g (−)-gallocatechin, 0.56 g (+)-catechin, 0.46 g (−)-epicatechin gallate, 0.16 g (−)-gallocatechin gallate and 0.01 g (−)-catechin gallate in a 100-g dry weight basis. In the experiment, instant green tea leaves in a bag (4 g) were infused with boiling water (400 mL, a serving portion) at 95 °C for 5 min. Sugars (50 g sucrose or 50 g isomaltulose) were added into the green tea beverage (400 mL).

### 2.2. Subjects

The sample size was calculated according to Torronen et al., considering the postprandial of glycemic response as the main variable [31]. A statistical power of 90% and an expected difference of 95% in the baseline values were adopted to form a total sample of at least 14 individuals. A total of eighteen subjects (aged 18–35 years old) were recruited from the local community through poster advertisement and flyers. The subjects were screened in terms of anthropometry (BMI ranged 18.5–22.9 kg/m^2^, % body fat <20% in men and <30% in women and waist circumference ≤90 cm in men and ≤80 cm in women), blood pressure <140/90 mmHg and blood chemistry (fasting plasma glucose level ≤100 mg/dL, total cholesterol <200 mg/dL, LDL-cholesterol <150 mg/dL, triglyceride <150 mg/dL, blood creatinine level ranging from 0.7–1.4 mg/dL and alanine aminotransferase (ALT) <40 IU/L). Participants were also excluded if they met the criteria for any evidence of physical illness, smoking, heavy drinking, history of chronic diseases, allergy, gastrointestinal pathologies (e.g., short bowel syndrome) and current use of drugs and food supplements. The study protocol was approved by the Ethics Review Committee for Research Involving Human Research Subjects, Health Science Group, Chulalongkorn University (No. 135/56). All subjects provided their written informed consent to participate in this study.

### 2.3. Study Design

The study was designed as a randomized, single-blinded, five-visit crossover study with a two-week washout period. At each experimental visit, after a 12-hour overnight fast, the subjects consumed the following beverage: (1) 50 g sucrose in 400 mL of water; (2) 50 g isomaltulose in 400 mL of water; (3) 400 mL of green tea; (4) 50 g sucrose in 400 mL of green tea; or (5) 50 g isomaltulose in 400 mL of green tea; within 5 min from the starting time. Blood samples were collected before and after 15, 30, 45, 60, 90 and 120 min of administration. During the experimental period, the subjects were instructed to avoid phenolic-rich foods (e.g., tea, coffee, fruit juices, berries, fruit, chocolate, etc.), high-antioxidant diets, alcoholic beverages and excessive exercise within a week before each visit. In addition, the subjects were asked to provide their food records and physical activity questionnaire at every visit.

### 2.4. Blood Collection

Blood samples were collected into tubes containing anticoagulants from an intravenous catheter inserted into a forearm vein. Blood samples were centrifuged at 3000 rpm for 15 min at 4 °C. Plasma samples were kept in a microtube and stored at −20 °C until analysis. Plasma glucose and insulin were analyzed using a glucose oxidase method (enzymatic colorimetric kits, GLUCOSE liquicolor, Human GmbH, Wiesbaden, Wiesbaden, Germany) and insulin human ELISA kit (BQ kits, San Diego, CA, USA), respectively.

### 2.5. The Ferric Reducing Ability of Plasma 

The ferric reducing ability of plasma was performed according to a previous study with slight modification [32]. In brief, the FRAP reagent was freshly prepared and warmed at 37 °C by mixing the following solution: (1) 0.3 M sodium acetate buffer solution (pH 3.6); (2) 10 mM 2,4,6-tripyridyl-1-5-triazine (TPTZ) in 40 mM HCl solution; and (3) 20 mM FeCl_3_ solution at the ratio of 10:1:1 (*v/v/v*), respectively. Plasma (10 µL) was incubated with 90 µL of FRAP reagent in a microplate for 30 min at room temperature in the dark. After that, the mixture measured the level of absorbance at the wavelength 595 nm with a spectrophotometer. The FRAP values were calculated by using a calibration standard curve of FeSO_4_ (0–2000 µM).

### 2.6. Lipid Peroxidation 

Plasma malondialdehyde (MDA) was quantified using a method based on the formation of thiobarbituric acid reactive substances (TBARS) and determined using fluorescence detection following a previous method with slight modification [33]. The plasma sample (60 µL) was incubated with 30 µL of 10% sodium dodecyl sulfate and 1.2 mL of TBA reagent (530 mg thiobarbituric acid in a mixed solution containing 50 mL of 20% acetic acid and 50 mL of 1 M NaOH). The mixtures were incubated in a heat block at 97 °C for 1 h. Following incubation, the mixtures were immediately removed from the heat block and placed in −20 °C for 10 min to stop the reaction. Next, the mixtures were centrifuged at 13,000 rpm for 10 min at 4 °C, and then, the supernatant was loaded into the microplate. The absorbance was measured using a fluorescence reader (Perkin Elmer^®^, Turku, Finland) at an excitation wavelength of 530 nm and an emission wavelength of 550 nm.

### 2.7. Statistical Analysis

For each test, the incremental data of plasma glucose, insulin, FRAP and MDA after consumption were analyzed using a repeated measurement ANOVA, followed by Duncan’s test at a significance level of *p* < 0.05. The incremental area under curves (iAUCs) were calculated according to the trapezoidal method. A one-way analysis of variance (ANOVA) followed by Duncan’s test for multiple comparison tests were performed to assess the differences between treatments (*p* < 0.05).

## 3. Results

### 3.1. Subjects

**Figure 1:**
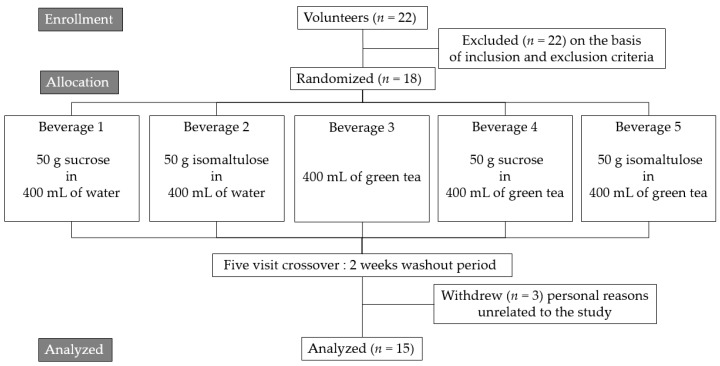
Flowchart for a randomized, single-blinded, five-visit crossover study.

Twenty-two subjects were recruited for this study according to the flowchart (Figure 1). Four subjects were excluded from the study following the basic inclusion and exclusion criteria of the study. The eighteen remaining subjects were randomly assigned into five groups. Three subjects withdrew after the first week due to reasons unrelated to the study. Fifteen subjects completed the study, including eight women and seven men. The baselines characteristics of the fifteen subjects are shown in Table 1. Furthermore, all subjects were instructed to maintain unchanged their lifestyles or physical behavior during the experimental period.

### 3.2. Glycemic Response

**Figure 2:**
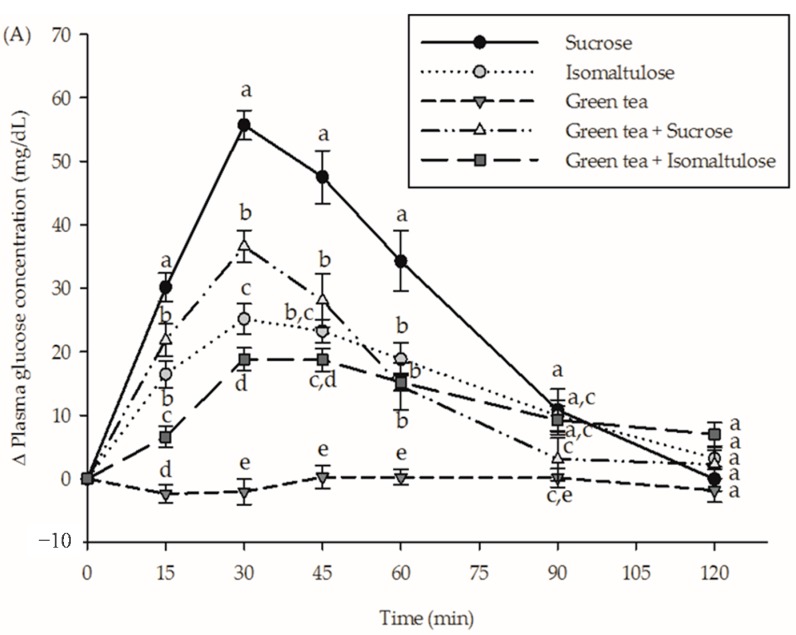

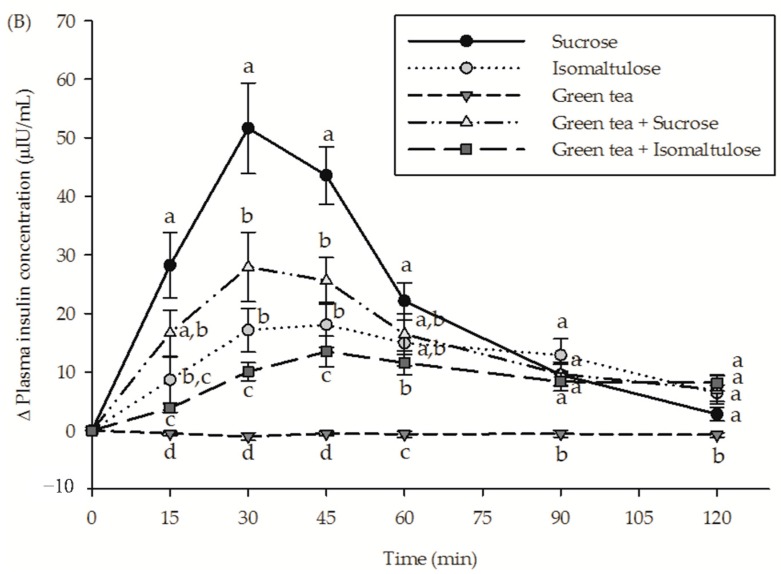
The incremental postprandial plasma (**A**) glucose concentration and (**B**) insulin concentration in healthy subjects after consumption of sucrose, isomaltulose, green tea, green tea plus sucrose and green tea plus isomaltulose (*n* = 15). Data are expressed as means ± SEM. Values not sharing the same superscript were significantly different between test groups in each time point (*p* < 0.05).

The incremental postprandial plasma glucose and insulin concentrations after the consumption of beverages are shown in Figure 2. Consumption of isomaltulose was found to significantly lower plasma glucose concentrations at 15, 30, 45 and 60 min and insulin concentrations at 15, 30 and 45 min as compared to the consumption of sucrose. The results showed that consumption of sucrose together with green tea significantly suppressed plasma glucose concentrations at 15, 30, 45, 60 and 90 min and insulin concentration at 30 and 45 min. In addition, the ingestion of green tea-containing isomaltulose caused a higher reduction of plasma glucose concentration (15 and 30 min) and insulin concentration (30 and 45 min) than isomaltulose alone.

**Figure 3:**
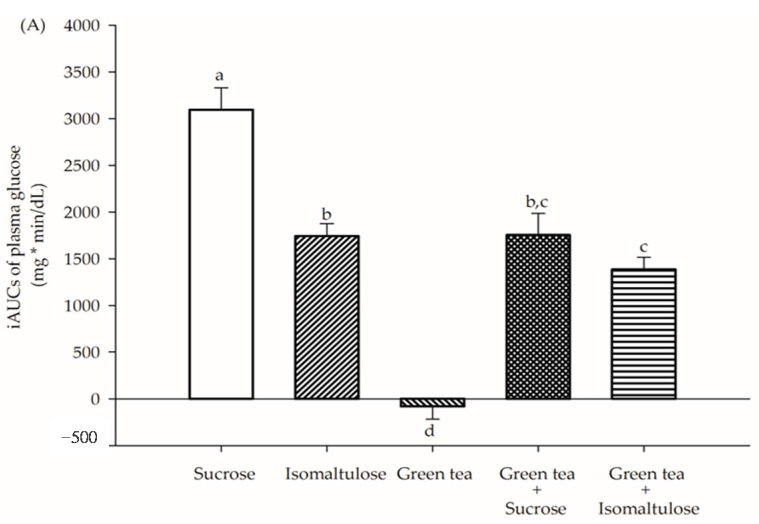

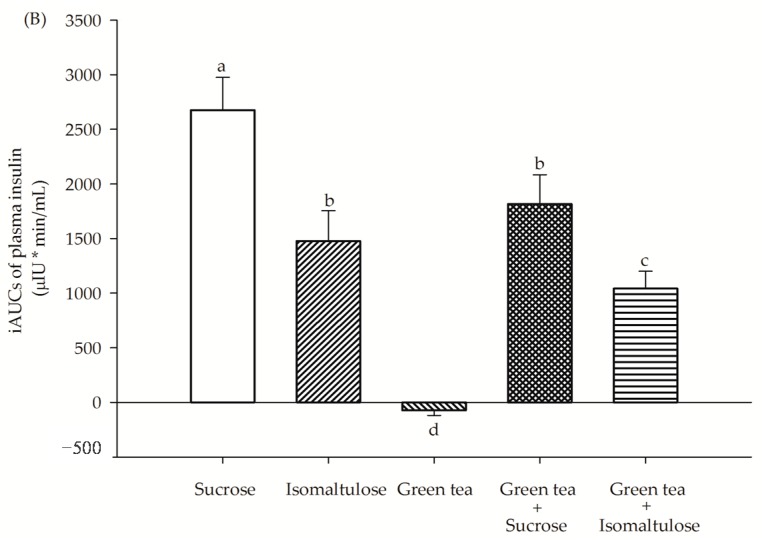
The incremental area under the curves (iAUCs) of plasma (**A**) glucose and (**B**) insulin concentration in healthy subjects after consumption of sucrose, isomaltulose, green tea, green tea plus sucrose and green tea plus isomaltulose (*n* = 15). Data are expressed as means ± SEM. Values not sharing the same superscript were significantly different between test groups (*p* < 0.05).

Postprandial iAUCs for glucose (43.3%) and insulin (42.0%) were largely reduced following the ingestion of isomaltulose compared to sucrose, respectively (Figure 3). Consumption of green tea-containing sucrose solution had lower iAUCs for glucose (43.4%) and insulin (32.1%) when compared to the sucrose solution. In addition, green tea-containing isomaltulose significantly reduced the iAUCs of glucose (20.9%) and insulin concentration (37.7%) when compared to isomaltulose.

### 3.3. Antioxidant Capacities

**Figure 4:**
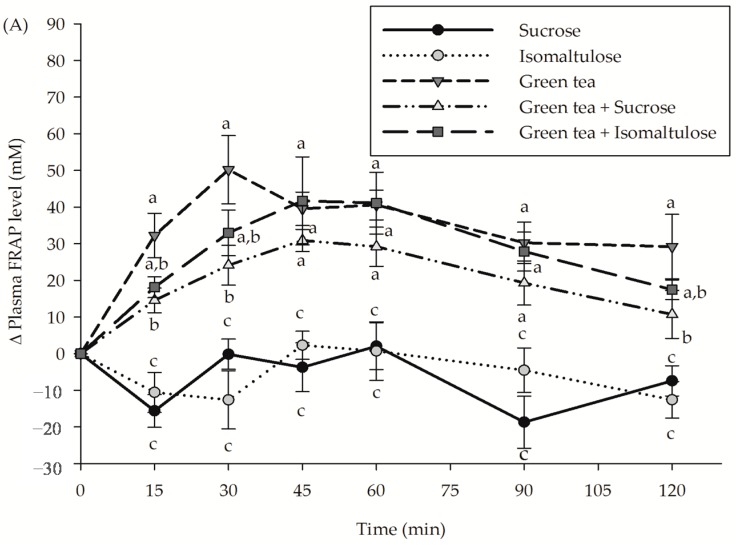

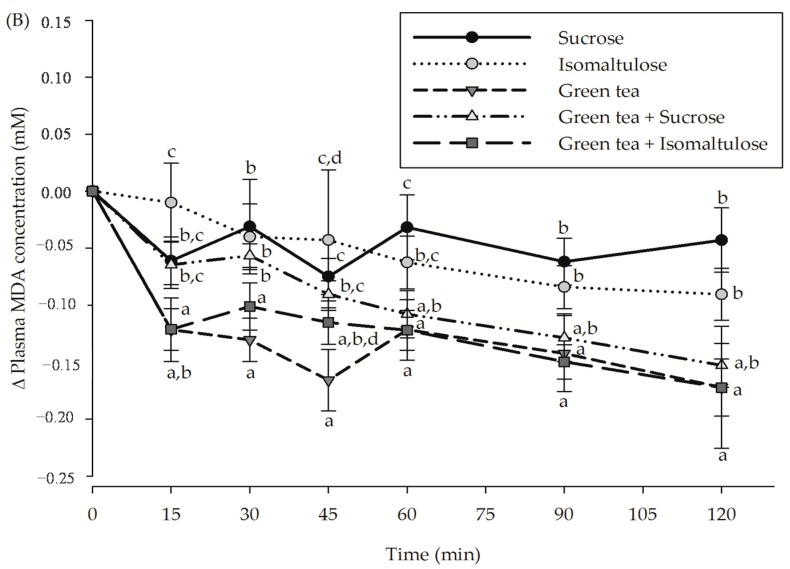
The incremental postprandial plasma (**A**) FRAP level and (**B**) malondialdehyde (MDA) concentration in healthy subjects after consumption of sucrose, isomaltulose, green tea, green tea plus sucrose and green tea plus isomaltulose (*n* = 15). Data are expressed as means ± SEM. Values not sharing the same superscript were significantly different between test groups in each time point (*p* < 0.05).

The incremental postprandial plasma FRAP and MDA concentrations of treatments are shown in Figure 4. Both sucrose and isomaltulose showed a slight decrease in postprandial plasma FRAP level. Consumption of green tea increased the plasma FRAP level during the experimental period; whereas the addition of sucrose to the solution caused a reduction in plasma FRAP level at 15, 30 and 90 min. In contrast, consumption of isomaltulose together with green tea maintained an increase in the postprandial plasma FRAP level during the study. The findings also demonstrated that sucrose and isomaltulose slightly reduced postprandial plasma MDA concentration during 2 h of consumption. In comparison with sucrose and isomaltulose, consumption of green tea resulted in a higher reduction of plasma MDA concentration. This effect declined with the combination of green tea with sucrose at 15 min and 30 min. In the meantime, replacing sucrose with isomaltulose slightly suppressed green tea-induced reduction of plasma MDA concentration at 15 min and 30 min.

**Figure 5:**
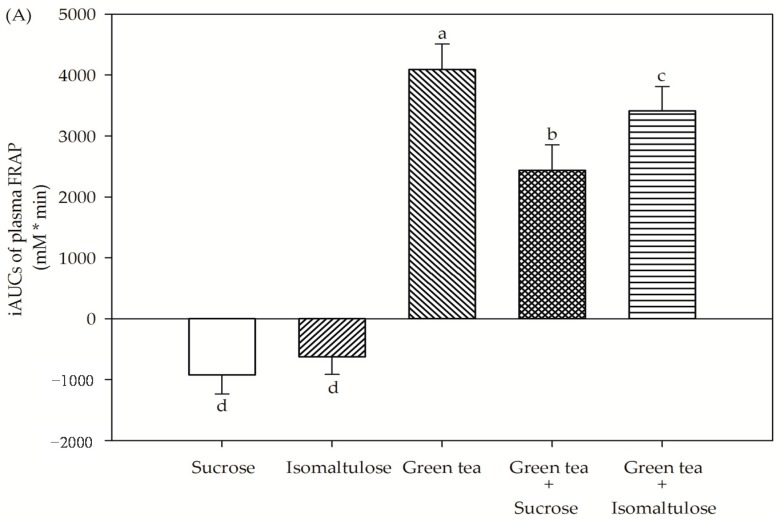

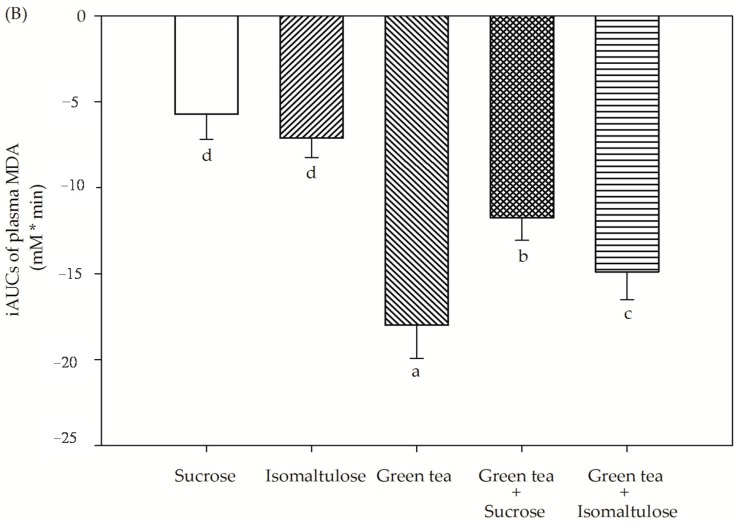
The incremental area under the curves (iAUCs) of plasma (**A**) FRAP and (**B**) MDA concentration in healthy subjects after consumption of sucrose, isomaltulose, green tea, green tea plus sucrose and green tea plus isomaltulose (*n* = 15). Data are expressed as means ± SEM. Values not sharing the same superscript were significantly different between test groups (*p* < 0.05).

The iAUCs of plasma FRAP and MDA concentrations are shown in Figure 5. Ingestion of green tea demonstrated the highest iAUC of plasma FRAP among all treatments. However, the iAUCs of plasma FRAP were significantly reduced with the consumption of green tea together with sucrose (40.4%) and isomaltulose (28.6%), respectively. At the same time, sucrose and isomaltulose attenuated green tea-induced reduction of iAUCs of plasma MDA by 34.7% and 17.2%, respectively.

## 4. Discussion

Previous studies have shown the antihyperglycemic activity of tea in healthy subjects [26,34]. Flavonoids, including catechin and its derivatives, exert effects on the reduction of postprandial glucose concentration in postmenopausal women [25]. Our findings are consistent with previous studies that the consumption of green tea together with sucrose reduces postprandial glucose and insulin concentration. The possible mechanisms of flavonoid-enriched green tea for reducing postprandial glucose include the inhibition of α-glucosidase activity, intestinal sodium-glucose co-transporter-1 (SGLT-1) and glucose transport-2 (GLUT-2) [35,36,37]. Furthermore, the flavins and catechins preferentially inhibited maltase rather than sucrose [38]. A recent study also revealed that epicatechin gallate competitively inhibited the glucose uptake through SGLT-1 [35]. In this connection, the postprandial glucose-lowering effects of flavonoid-enriched green might be associated with the inhibitory activity against the α-glucosidase and intestinal glucose transporter. Consistent with previous studies, replacing sucrose with isomaltulose markedly reduced postprandial plasma glucose and insulin concentrations in healthy and overweight subjects [13,39]. For example, consumption of 140 g cookies and 250 mL of liquid containing 50 g isomaltulose was found to be more effective at reducing postprandial plasma glucose and insulin concentration than sucrose in overweight subjects [39]. The suppression of hyperglycemia might be due to the slow digestion and absorption rate of isomaltulose in the small intestine [11,12]. When the subjects received isomaltulose plus green tea, a magnitude reduction of postprandial glucose and insulin concentration was achieved during the experimental period as compared to isomaltulose. The significant effect was mainly observed at 15 and 30 min. In addition to the isomerization of sucrose, isomaltulose can be slowly digested by α-glucosidase (isomaltulase). Its digestive products (glucose and fructose) are absorbed into the enterocytes by SGLT-1 and GLUT5, respectively. Finally, GLUT2 in enterocytes also aids in the transport of glucose and fructose into the blood circulation. In light of this, the inhibition of α-glucosidase activity could explain the interaction between tea catechins and isomaltulose in the gastrointestinal tract. It is possible that tea catechins might suppress the digestion of isomaltulose by inhibiting isomaltulase and/or slower absorption of the liberated glucose and fructose. Impaired digestion and/or absorption results in the reduced peak of postprandial glucose and insulin concentration.

The consumption of antioxidant-containing foods has been implicated to play a possible role in the prevention of chronic and age-related diseases [40]. Antioxidants reduce free radical-induced damage to protein, lipid and DNA, thus leading to the prevention of oxidative injury [41]. An increase in plasma FRAP level reflects the dietary intake of antioxidants and indicates the level of antioxidants in blood circulation [32]. Malondialdehyde (MDA) is commonly used as a marker of lipid peroxidation. In blood circulation, the accumulation of MDA contributes to modifying the structure of low-density lipoprotein (LDL), one of the main initiators of atherogenesis [42]. In the present study, the consumption of green tea increased plasma FRAP level concomitant with the reduction of plasma MDA concentration. Similar findings have been reported in other human studies. In a crossover study with 10 healthy subjects, green tea resulted in a 4% increase in FRAP after 40 min of consumption [43]. A greater increase in plasma FRAP was observed between 30 and 60 min after healthy volunteers drank green tea (2 g tea solids in 300 mL water) [44]. The decrease in plasma MDA concentration was seen after the consumption of green tea [45,46]. This is in accordance with previous studies in that the consumption of green tea resulted in a significant rise in plasma antioxidant activity associated with an increase in the concentration of plasma catechins [40]. Several studies support that tea catechins demonstrated antioxidant activity by scavenging free radicals and chelating redox-active transition metal ions [21,23,47]. Previous studies provided the FRAP value of tea catechins, and the order was as follows: ECG > EGCG ≈ GCG > GC ≈ EGC > C ≈ EC [48]. Furthermore, tea catechins prevented lipid peroxidation both in in vitro and in vivo models [22,23,49]. The acute rise of plasma FRAP, together with the reduction of the plasma MDA concentration, may be related in part to the presence of tea catechins. When sucrose was added to green tea, an increase in plasma FRAP level and a reduction of plasma MDA concentration were attenuated. In contrast to sucrose, isomaltulose did not impact the alteration of plasma FRAP and MDA concentration. The mechanisms by which sucrose interferes with plasma FRAP and MDA concentrations remain unclear. It is possible that sucrose remaining within the intestinal lumen may serve to interfere with the absorption of catechin and its derivatives in association with the reduction of the plasma FRAP level. A model of (−)-epicatechin gallate (ECG) absorption in the enterocyte has been recently proposed. ECG is actively absorbed across the apical membrane by monocarboxylate transporter-1 (MCT-1) [50]. Moreover, the uptake of ECG was sodium independent and pH gradient dependent. The interaction between sucrose and MCT-1 could occur in the gut; this phenomenon may be of major importance in achieving a significant reduction of the tea catechins’ uptake into blood circulation. Additional experiments are required to determine the effect of sucrose and isomaltulose regarding the absorption of tea catechins in intestinal cell models. Several studies provide a correlation between the intake of the dietary total antioxidant capacity and the incidence of chronic diseases and mortality. Cohort studies investigated the inverse associations between dietary total antioxidant capacity (TAC) and stroke and myocardial infraction [51], reporting that a diet high in TAC, as measured by the Trolox equivalent antioxidant capacity (TEAC) and FRAP, has been inversely associated with pancreatic cancer risk [52]. Additionally, FRAP dietary equivalent intake was inversely associated with mortality from cancer and cardiovascular diseases [53]. The sustained elevation of total antioxidant status has been associated with the lower incidence of cardiovascular diseases (CVDs) in populations who regularly consumed red wine [54]. Consumption of green tea together with sucrose should be raised as a concern. Although drinking green tea together with sucrose suppressed a rise in postprandial plasma glucose concentration, it also caused the lower antioxidant capacity of plasma. This evidence may be a limitation regarding the inability to reach the sustained level of total antioxidant capacity for chronic disease prevention. The beneficial effects of green tea can sustain plasma antioxidant capacity concomitant with the suppression of postprandial glucose when consumed with isomaltulose. A limitation of this study is the small number of subjects enrolled. As the participants were healthy, there is a lack of an outcome associated with the alteration of antioxidant status after the intake of green tea together with sucrose or isomaltulose.

## 5. Conclusions

The consumption of green tea enhances the reduction of postprandial glucose and insulin concentration when the subjects consumed it with isomaltulose. Replacing sucrose with isomaltulose in green tea improves the plasma antioxidant capacity, as measured by the level of FRAP and the concentration of MDA. The evaluation of the long-term effects of green tea and isomaltulose deserves further attention.

## Figures and Tables

**Table 1:** Baseline characteristics of fifteen subjects (8 women and 7 men).

Parameters	Mean ± SEM
Age (years)	23.5 ± 0.7
Weight (kg)	21.0 ± 0.4
BMI (kg/m^2^)	22.6 ± 1.4
% Body fat	
Women	22.6 ± 1.4
Men	13.6 ± 1.1
Waist circumference (cm)	
Women	69.9 ± 2.4
Men	80.0 ± 2.2
Systolic blood pressure (mmHg)	115.3 ± 1.9
Diastolic blood pressure (mmHg)	71.9 ± 2.3
Fasting glucose (mg/dL)	81.5 ± 2.3
Total cholesterol (mg/dL)	186.5 ± 3.2
LDL-cholesterol (mg/dL)	119.0 ± 5.2
Triglyceride (mg/dL)	76.5 ± 6.3
Creatinine (mg/dL)	0.9 ± 0.1
Alanine aminotransferase or ALT (U/L)	10.7 ± 1.3
